# Anesthesia management of a patient with giant goiter, multiple metastases, and airway stenosis: a case report

**DOI:** 10.3389/fmed.2026.1888635

**Published:** 2026-08-26

**Authors:** Liuxu Yao, Junbiao Fang, Yiyang Hu, Weiying Xie, Wen Zhai

**Affiliations:** 1 Center for Rehabilitation Medicine, Department of Anesthesiology, Zhejiang Provincial People's Hospital (Affiliated People's Hospital, Hangzhou Medical College), Hangzhou, Zhejiang, China; 2 Research Institute of Anesthesiology and Perioperative Medicine, Hangzhou Medical College, Hangzhou, Zhejiang, China

**Keywords:** airway obstruction, anesthesia management, awake fiberoptic-guided tracheal intubation, giant goiter with multiple metastases, meticulous perioperative management

## Abstract

Patients with giant papillary thyroid carcinoma (PTC) and extensive metastases involving the anterior mediastinum and both lungs face a high risk of severe airway obstruction and ventilation difficulties during anesthesia induction. We report an 18-year-old female with a giant PTC causing critical tracheal compression and extensive metastases. Awake fiberoptic intubation was successfully performed while maintaining spontaneous ventilation. Total thyroidectomy combined with thoracotomy lasted for 10 h, and the blood loss during the operation was about 4,500 mL. After operation, the patient was successfully extubated, and no immediate respiratory complications occurred. However, complications such as acute respiratory distress syndrome (ARDS), acute kidney injury and tracheal fistula occurred in the follow-up treatment. After comprehensive treatment, the patient recovered and was discharged two months later. This case highlights that meticulous perioperative management can provide a safe and effective approach for patients with giant thyroid tumors, extensive mediastinal and pulmonary metastases, and compromised airway integrity.

## 1 Introduction

Papillary thyroid carcinoma (PTC) is the most common type of thyroid malignancy, accounting for approximately 85% of all thyroid cancers; however, distant metastasis is relatively uncommon (1). A giant goiter extending to the mediastinum may compress the airway and mediastinal vessels, potentially leading to serious perioperative complications, including severe hypoxia, vascular events, and cardiac arrest (2, 3). These patients are particularly susceptible to critical airway obstruction and ventilation difficulties during anesthesia induction. Therefore, rapid establishment of a secure airway while ensuring adequate surgical exposure is essential. Here, we report a rare case of giant PTC with extensive metastases involving the anterior mediastinum and both lungs, accompanied by severe tracheal compression, and discuss the perioperative anesthesia management strategies for such high-risk patients.

## 2 Case presentation

**Figure 1:**
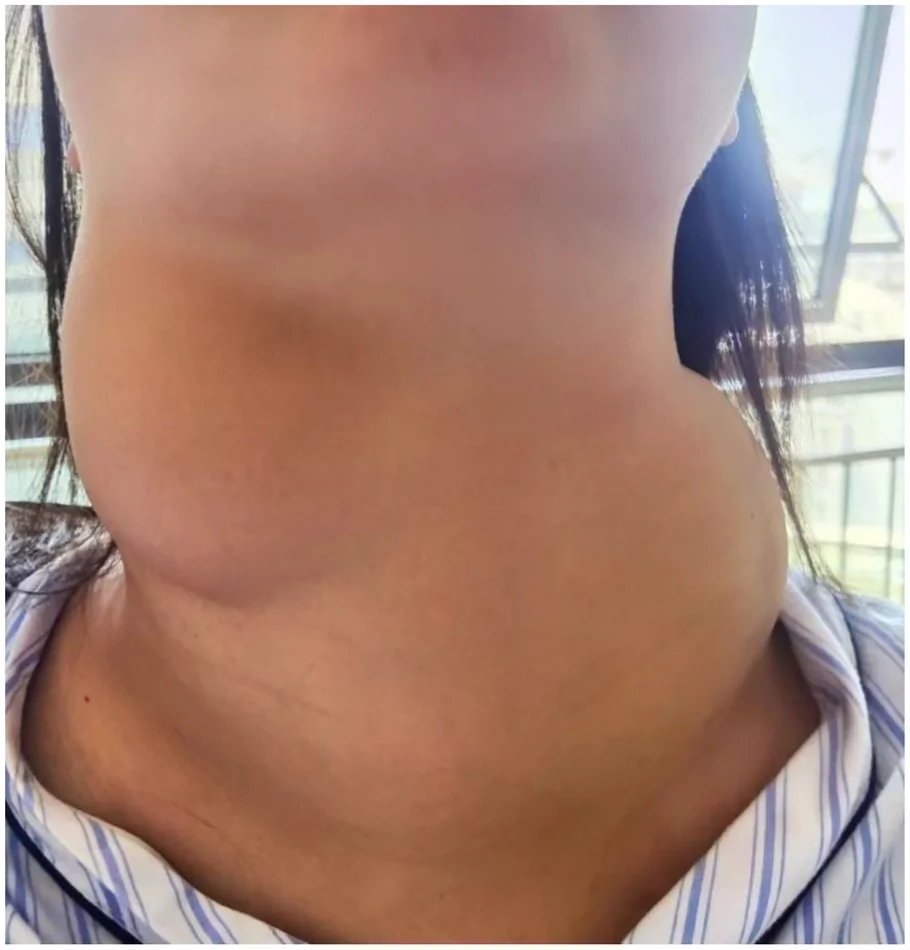
Physical examination revealed multiple cervical masses.

**Figure 2:**
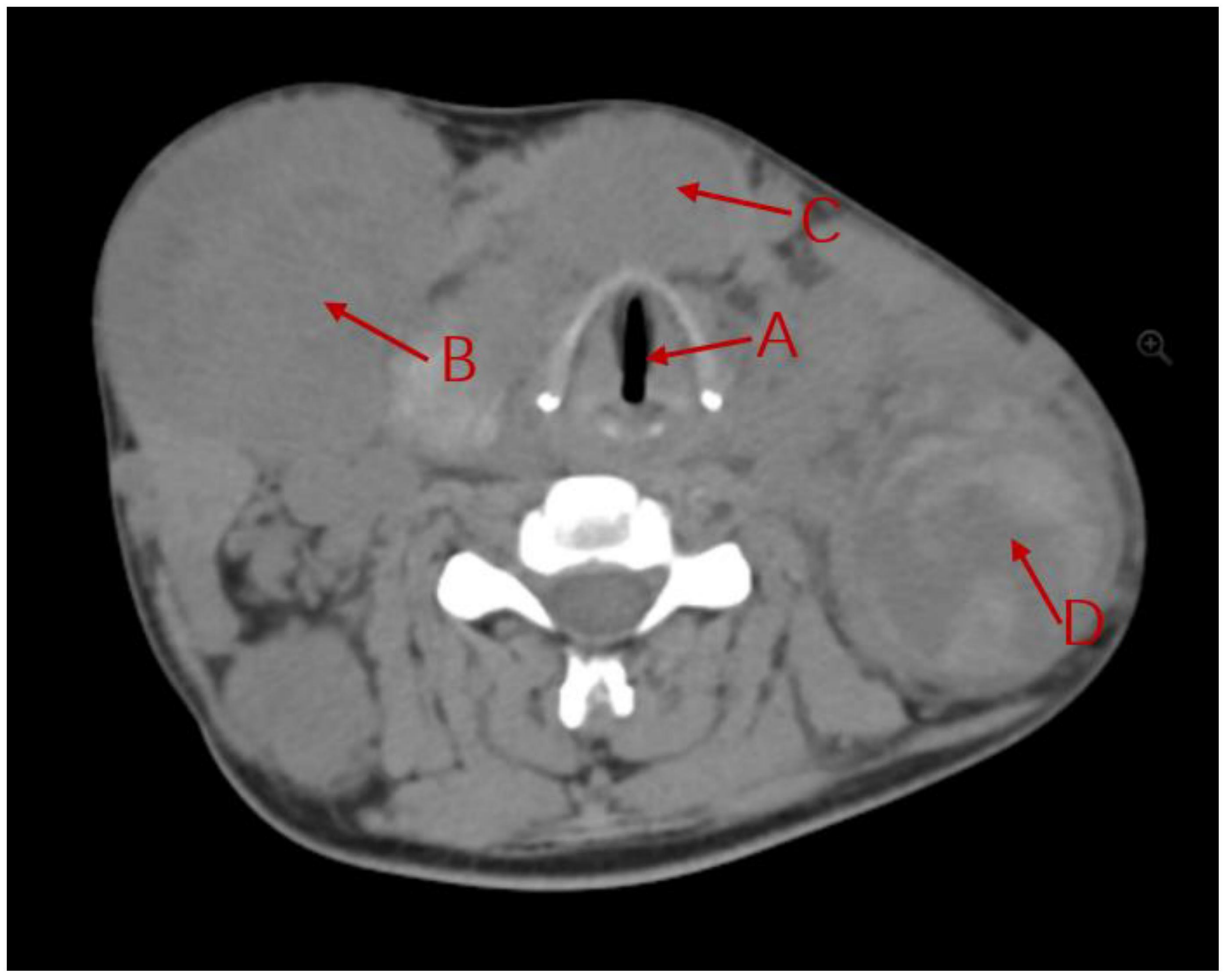
**(A)** Tracheal stenosis. **(B–D)** Enlarged thyroid gland and cervical lymphadenopathy.

**Figure 3:**
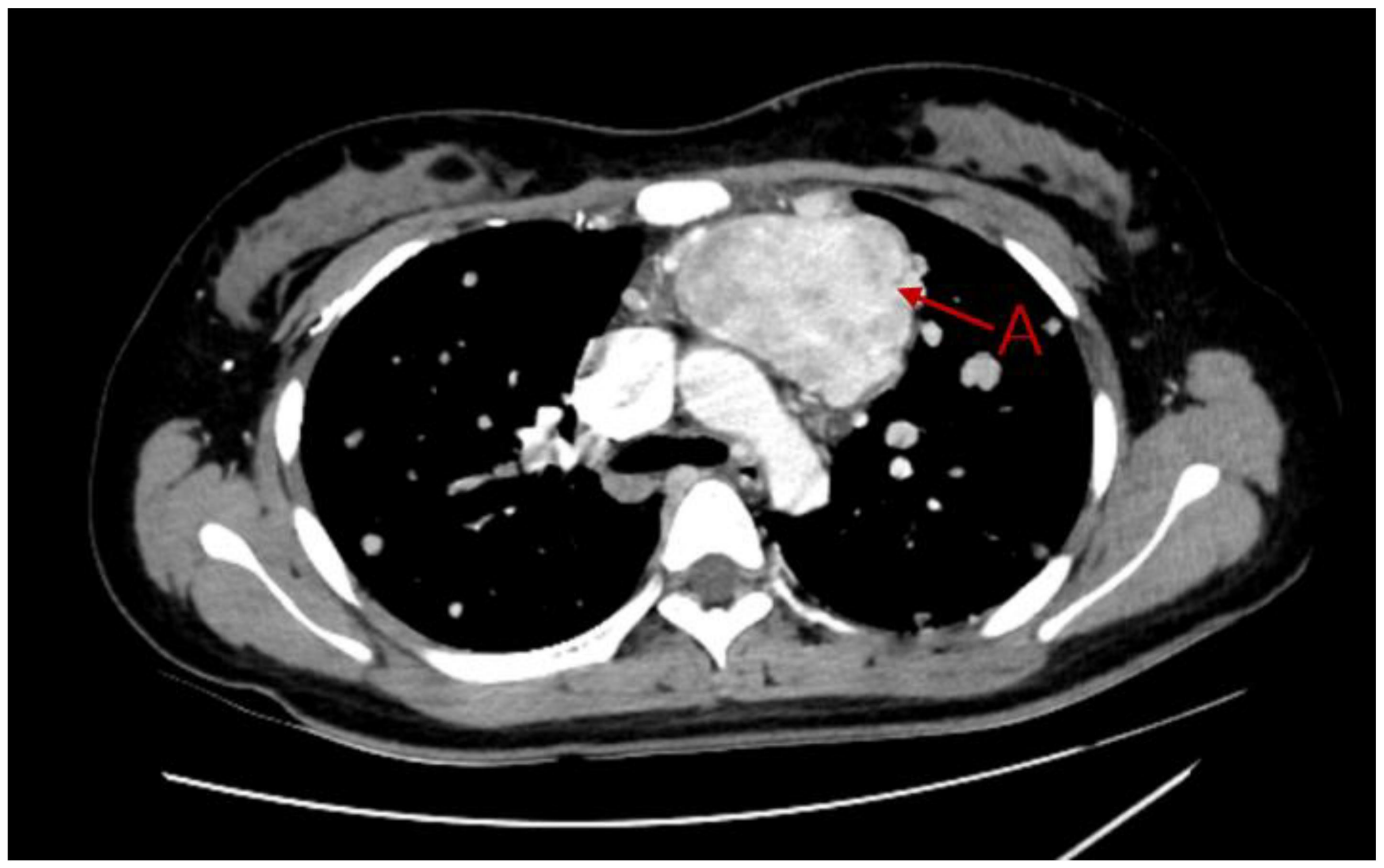
Anterior mediastinal mass.

**Figure 4:**
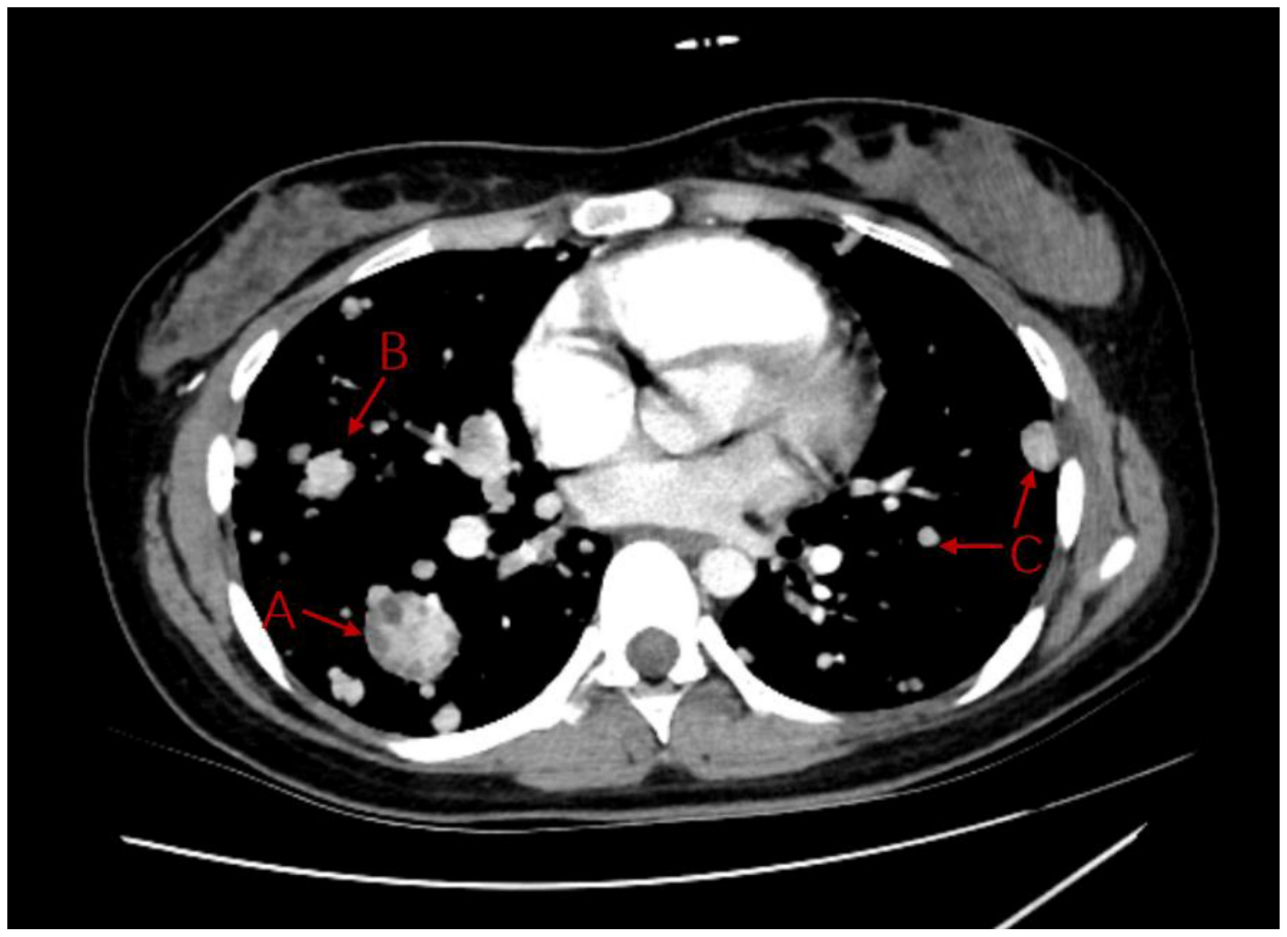
**(A–C)** Pulmonary metastatic nodules.

An 18-year-old female patient with a more than 10-year history of a progressively enlarging neck tumor was admitted to the hospital because of worsening dyspnea and a sensation of suffocation. The mMRC's (modified Medical Research Council, mMRC) dyspnea is classified as Grade 3. Positional changes markedly aggravated suffocation in the supine and neck-reclining positions, necessitating the lateral decubitus, high-pillow, or semi-supine position. No wheezing, hoarseness or dysphagia. Physical examination on admission revealed multiple large cervical masses. The neck appeared markedly thickened with tracheal deviation and poor mobility on palpation. The inter-incisor distance was approximately 4 cm, Mallampati grade II, thyromental distance (TMD) was approximately 7 cm. and limited neck extension (due to the pulling sensation of the tumor). The right upper neck mass measured approximately 10 cm, the posterior right neck mass measured approximately 8 cm, and a large mass involving the left and midline neck measured approximately 15 cm in diameter (Figure 1). Contrast-enhanced CT of the head, neck, and chest revealed diffuse enlargement of the thyroid gland, measuring approximately 101 × 72 mm. Multiple enlarged cervical lymph nodes were also identified, with a size of about 68 × 55 mm. In addition, metastatic lesions were identified in the anterior mediastinum and both lungs, accompanied by the invasion of airway wall. The anterior mediastinal mass measured approximately 70 × 52 mm, and multiple diffuse pulmonary nodules were observed bilaterally. The largest pulmonary lesion was located in the posterior basal segment of the right lower lobe and measured approximately 32 × 27 mm. The trachea was markedly compressed with significant luminal narrowing (Figures 2–4). Laboratory investigations revealed a markedly elevated thyroglobulin level (1,500 μg/L; reference range: 1.6–59.9 μg/L). Routine blood tests, as well as liver and renal function parameters, were within normal limits.

To ensure perioperative safety and optimize surgical outcomes, a multidisciplinary team (MDT) consultation involving thyroid surgeons, cardiothoracic surgeons, senior anesthesiologists, intensivists, and transfusion medicine specialists was conducted preoperatively to develop a comprehensive surgical and anesthetic management strategy. Surgeons experienced in emergency tracheotomy, together with the extracorporeal membrane oxygenation (ECMO) team, high-frequency jet ventilation system (HFJV) standby, remained on standby throughout the procedure to manage potential airway emergencies. ECMO support was planned to be initiated under the following conditions: (1) peak airway pressure >35 cmH_2_O with failure to achieve adequate ventilation; (2) bronchoscopic confirmation of dynamic tracheal collapse; (3) SpO_2_ < 90% refractory to conventional interventions; (4) SBP < 60 mmHg for >2 min despite vasopressor support (norepinephrine ≥0.3 μg/kg/min). If ECMO initiation became necessary, experienced ECMO specialists would perform femoral artery and venous cannulation under local anesthesia, followed by the initiation of venoarterial (V-A) extracorporeal membrane oxygenation support. Given poor exposure of the tracheal rings and anticipated difficulty of cricothyroid membrane puncture, awake fiberoptic bronchoscope (FOB)-guided tracheal intubation was planned with topical oropharyngeal anesthesia combined with intravenous sedative and analgesic agents, while maintaining spontaneous ventilation. Based on CT findings, a 6.5-mm internal diameter (ID) endotracheal tube (ETT) (Kehui Company) was selected to pass through the narrowest tracheal segment, measured as 4.2 mm in width and 3.7 cm in length on CT. The stenosis was located approximately 5.23 cm from the glottis and 9.6 cm from the carina. Smaller-ID ETTs (4.0, 4.5, 5.0, 5.5, and 6.0 mm) were prepared as backups. The tube was successfully advanced beyond the stenotic segment caused by the giant goiter, and the tube tip was positioned 21 cm from the incisors.

On the morning of surgery, the patient underwent nebulization with 2% lidocaine for 20 min before entering the operating room. Upon arrival, vital signs were stable, with a blood pressure of 118/65 mm Hg, heart rate of 88 beats/min, and oxygen saturation (SpO_2_) > 95%. Additional topical anesthesia was achieved by spraying 2% lidocaine (100 mg in total) onto the oropharynx to further reduce airway irritation during intubation. Patients were pre-oxygenated with 10 L/min 100% oxygen mask for more than 5 min in the semi-supine position until the oxygen saturation (SpO2) exceeded 100%. Intravenous dexmedetomidine (30 μg infused over 15 min) and sufentanil (5 μg) were administered to improve tolerance to awake intubation and minimize discomfort. After clinical assessment of adequate oxygenation (SpO_2_ 100% on simple face mask at 10 L/min for 5 min) and stable spontaneous ventilation (regular breathing, symmetrical chest movement, no distress), awake tracheal intubation under spontaneous breathing was successfully performed using a FOB with video laryngoscopic assistance. Once the tip of the endotracheal tube had passed through the narrowest segment of the airway, intravenous propofol (150 mg), rocuronium (30 mg), and sufentanil (20 μg) were immediately administered. Sevoflurane inhalation anesthesia [0.3 minimum alveolar concentration (MAC)] was initiated to deepen anesthesia. Fiberoptic bronchoscopy performed before and after intubation confirmed both successful traversal of the endotracheal tube through the narrowest portion of the trachea and distal positioning of the tube tip beyond the stenotic segment, with no evidence of dynamic airway collapse or tracheomalacia. Correct tube placement was further verified by end-tidal CO_2_ monitoring and airway pressure measurements. Mechanical ventilation was maintained with a tidal volume of 420 mL, respiratory rate of 12 breaths/min, oxygen concentration of 60%, and airway pressure of 16 mmHg, all of which remained within acceptable ranges. After surgical positioning was completed, fiberoptic bronchoscopy was repeated to reconfirm appropriate endotracheal tube positioning.

**Figure 5:**
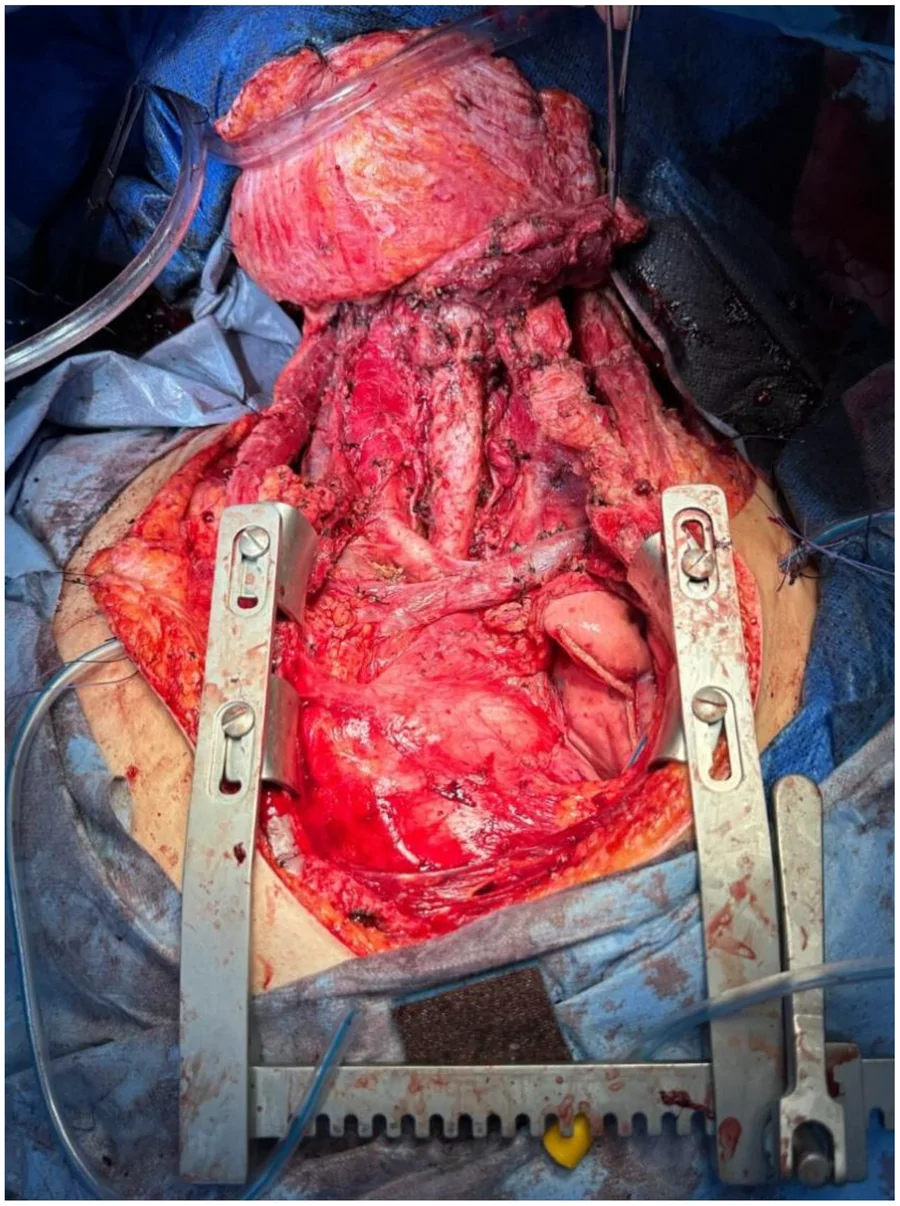
Median sternotomy for resection of the mediastinal tumor.

**Figure 6:**
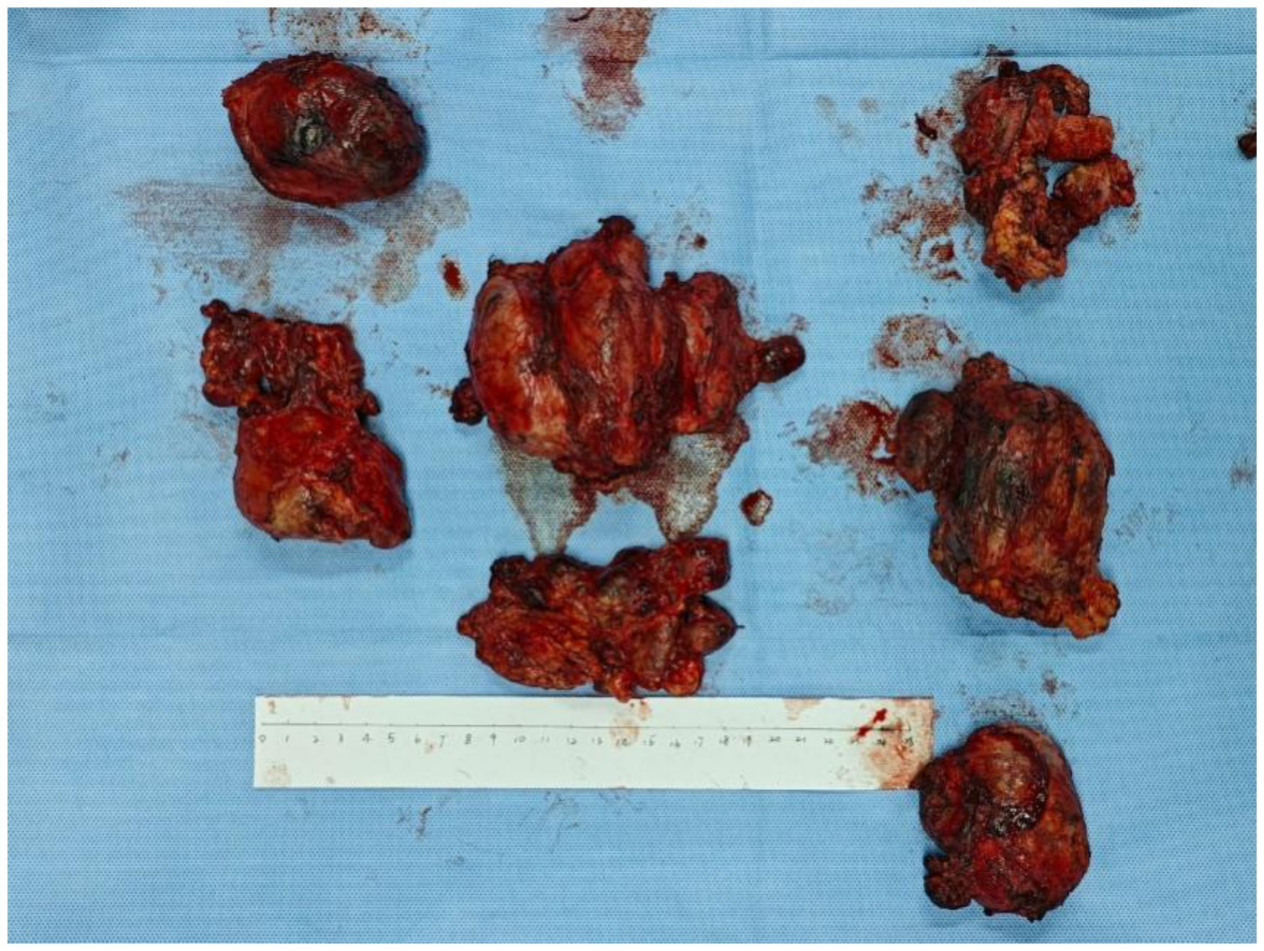
Resected tumor specimen.

Anesthesia was maintained with 1% sevoflurane, propofol (4–6 mg/kg/min), remifentanil (0.1–1 μg/kg/min), and intermittent rocuronium supplementation (10 mg every 40 min). A cell salvage system with LDF was prepared before skin incision and used intraoperatively for autologous blood recovery and reinfusion. Intraoperatively, the bilateral thyroid glands were found to be diffusely enlarged, extending superiorly to the hyoid bone and inferiorly into the retrosternal region. The mass was closely related to the recurrent laryngeal nerves, trachea, and esophagus. After approximately 6 h of surgery, a median sternotomy extending from the sternal notch to the xiphoid process was performed. Exploration revealed that the mediastinal tumor was densely adherent to the bilateral pleural cavities and innominate vein, with severe adhesion to the left lung lobe (Figure 5). During resection of the mediastinal tumor, thoracic surgeons cooperated closely with head and neck surgeons. The patient's preoperative hemoglobin was 140 g/L, which was within the normal range. However, transection of the innominate vein led to massive hemorrhage, with blood loss exceeding 2,000 mL within a short period. Consequently, the hemoglobin level rapidly decreased to a critical low of 32 g/L, accompanied by a precipitous decline in blood pressure. Hemodynamic support was achieved through continuous norepinephrine infusion (0.01–0.1 µg/kg/min), intermittent phenylephrine boluses (10 µg), and aggressive fluid resuscitation. Intraoperative autologous blood reinfusion was therefore initiated. The operation was successfully completed after approximately 10 h, with complete resection of both the cervical and mediastinal masses (Figure 6). Total intraoperative blood loss was approximately 4,500 mL. The patient received 8 units of packed red blood cells, 1,750 mL of autologous blood reinfusion, 1,340 mL of plasma transfusion, 600 IU of prothrombin complex concentrate, and 1 g of fibrinogen concentrate. Total urine output was 4,000 mL. Serial arterial blood gas analyses confirmed that serum lactate remained within normal limits throughout the procedure, indicating adequate tissue perfusion despite transient hypotension. By the end of the surgical procedure, after aggressive volume resuscitation and multiple blood product transfusions, the hemoglobin had risen to 79 g/L. Given the extensive surgical dissection and the potential risk of recurrent laryngeal nerve injury, tracheal injury, and postoperative hemorrhage, the patient was transferred to the intensive care unit (ICU) with the endotracheal tube in place for continued monitoring and respiratory support. Before leaving the operating room, fiberoptic bronchoscopy was performed to confirm proper endotracheal tube positioning and relief of airway compression. The patient was extubated on postoperative day 2. Postoperatively, the patient developed several significant complications that required intensive management. On the third day after extubation, the patient was diagnosed with severe ARDS secondary to massive transfusion-related lung injury, strictly meeting the Berlin Definition criteria (PaO_2_ 57 mmHg on high-flow nasal cannula with FiO_2_ 70%, corresponding to a PaO_2_/FiO_2_ ratio of approximately 81 mmHg). The patient required reintubation and received invasive mechanical ventilation (pressure-controlled ventilation mode, PEEP 5–8 cmH_2_O), along with inhaled nitric oxide and prone positioning as adjunctive therapies. After two weeks of intensive respiratory support, a tracheostomy was performed to facilitate gradual weaning from mechanical ventilation. Concurrently, the patient developed acute kidney injury within 24 h postoperatively, with serum creatinine rising from a preoperative baseline of 50 µmol/L to 98 µmol/L, meeting KDIGO Stage 1 criteria. However, the patient remained non-oliguric with a 24-hour urine output of 2,000–3,000 mL, and renal function recovered to baseline within 48 h without requiring renal replacement therapy. Furthermore, a tracheal fistula developed as a delayed complication following tracheostomy, which was successfully managed with surgical repair after the patient's clinical condition had stabilized. Despite these significant postoperative challenges, the patient responded well to comprehensive multidisciplinary treatment and was successfully discharged after 2 months with satisfactory recovery. Histopathological examination confirmed PTC of mixed subtype (classical and follicular variants). Because of the extensive bilateral pulmonary metastases, surgical resection of the lung lesions was not feasible. The patient received iodine-131 treatment three times after the operation, long-term metrel treatment and regular follow-up examinations, and the condition was stable.

## 3 Discussion

In the present case, the giant thyroid mass caused severe tracheal compression and was associated with retrosternal extension and multiple pulmonary metastases. Consequently, the patient was at high risk for perioperative airway compromise, vascular injury, massive hemorrhage, and postoperative complications. The successful management of this patient depended on three key principles.

Comprehensive preoperative preparation served as the cornerstone of successful management, with the timely establishment of a multidisciplinary team representing the first critical step. A detailed perioperative management plan with clearly defined backup strategies was formulated according to the core management principle of difficult airway (4), to ensure structured responses to potential high-risk intraoperative events. During airway management, even minor stimulation could potentially trigger airway spasm or complete airway collapse. Therefore, intubation had to be completed successfully on the first attempt, with the distal end of the tube advanced beyond the stenotic segment. Recognizing this uncertainty, we incorporated two critical backup strategies into our anesthetic plan: HFJV and ECMO standby. HFJV was prepared as a rescue ventilation modality in the event of complete airway obstruction or inability to ventilate through the conventional endotracheal tube. V-A ECMO standby was arranged with our cardiothoracic surgical team, to be emergently instituted in the event of catastrophic airway compromise with failure of both conventional and jet ventilation. ECMO may serve as an important rescue strategy in patients with giant substernal goiters when airway patency cannot be safely maintained, even under preserved spontaneous ventilation (5, 6). Given that anesthetic considerations for mediastinal masses are highly dependent on specific anatomical relationships, individualized planning remains essential (7). In contrast to previously reported cases where perioperative preparations were limited to awake intubation alone or ECMO as a primary strategy without backup measures (8), our management incorporated a tiered, multimodal approach. Although this case did not activate ECMO in the end, it is of great reference value to emphasize ECMO backup in this case. The patient has tracheal compression and innominate vein invasion. ECMO is reserved and activated under certain conditions to provide safety for the patient.

Secondly, refined intraoperative managemen was critically important. Awake fiberoptic-guided tracheal intubation preserves spontaneous ventilation and upper airway muscle tone, thereby providing maximal safety for anticipated difficult airway management and facilitating subsequent operations (9). Dynamic airway evaluation using a FOB can directly guide clinical decision-making and is consistent with current recommendations for the management of anticipated difficult airway (10). Compared with traditional intubation techniques, awake fiberoptic-guided intubation provides superior visualization of the airway, enables real-time manipulation within complex anatomical structures, and reduces the risk of intubation failure or airway injury (11). More importantly, it facilitated advancement of the endotracheal tube beyond the stenotic segment. These advantages were clearly demonstrated in the present case, in which awake fiberoptic-assisted tracheal intubation was successfully performed. Previous studies have suggested that tracheal deviation observed on cross-sectional CT may not necessarily correlate with symptoms of tracheoesophageal compression (12). Furthermore, the airway may become compliant during spontaneous inspiration due to the cystic and soft-tissue characteristics of giant goiters, thereby allowing the endotracheal tube to traverse the stenotic segment and reach the distal trachea. This phenomenon may explain why successful intubation through the narrowest airway with a 6.5-mm endotracheal tube was possible in this patient. Postoperative airway evaluation following tumor resection also assisted in determining the optimal timing for staged extubation in the ICU, thereby reducing the risk of serious postoperative complications (13). However, airway management represented only one dimension of the perioperative challenge. In this case, the mediastinal tumor originated from the thyroid gland and exhibited severe adhesions to the pericardium and pleura, substantially increasing the risk of massive hemorrhage, vascular injury, pulmonary injury, and even cardiac arrest during surgery. Adequate preoperative blood preparation and intraoperative autologous blood transfusion were therefore critical for preventing hemorrhagic shock. Intraoperative recurrent laryngeal nerve monitoring can improve the detection of nerve injury, facilitate timely corrective measures, and reduce the incidence and severity of postoperative complications (14).

Ultimately, the exceptional nature of this case arose not from any single technical intervention, but from the rare convergence of multiple life-threatening complications throughout the perioperative continuum, including severe central airway compromise, catastrophic intraoperative hemorrhage requiring massive transfusion, and a sequential cascade of postoperative complications involving severe ARDS, AKI, and delayed tracheal fistula formation. The development of ARDS and AKI highlighted the complex interplay between hemorrhagic shock, transfusion-related immunomodulation, and end-organ injury. Notably, the non-oliguric nature of AKI in this patient was associated with a more favorable prognosis, consistent with previous evidence indicating that even mild AKI may significantly influence clinical recovery trajectories (15). The delayed tracheal fistula, an exceedingly rare complication with an estimated incidence of approximately 0.006% (16), was likely attributable to multifactorial mechanisms, including pre-existing tracheal wall compromise, surgical devascularization, and ischemic injury associated with prolonged mechanical ventilation. What distinguishes this case is the sequential and interdependent nature of these complications, in which each adverse event potentially contributed to the development of subsequent complications: massive hemorrhage necessitated extensive transfusion, which increased susceptibility to ARDS and AKI, while prolonged respiratory support required for ARDS management may have further contributed to tracheal fistula formation. This interconnected clinical trajectory provides an important lesson: in patients with giant goiters and mediastinal masses, perioperative risk extends far beyond the well-recognized threat of airway collapse during induction of anesthesia. Successful management requires not only expertise in difficult airway strategies but also proactive anticipation of downstream complications, close multidisciplinary collaboration, and continuous vigilance extending beyond the operating room.

In summary, patients with giant goiters associated with extensive metastatic disease and severe tracheal compression require proactive, meticulous, and multidisciplinary perioperative management to mitigate life-threatening complications and optimize clinical outcomes.

## Data Availability

The original contributions presented in the study are included in the article/Supplementary Material, further inquiries can be directed to the corresponding author.
